# Systemic regulation of mineral homeostasis by micro RNAs

**DOI:** 10.3389/fpls.2013.00145

**Published:** 2013-05-16

**Authors:** Julia Kehr

**Affiliations:** Department of Molecular Plant Genetics, University of HamburgHamburg, Germany

**Keywords:** nutrient, homeostasis, systemic, signaling, phloem, long-distance, transport

## Abstract

Plants frequently have to cope with environments with sub-optimal mineral nutrient availability. Therefore they need to constantly sense changes of ion concentrations in their environment. Nutrient availabilities and needs have to be tightly coordinated between organs to ensure a balance between uptake and demand for metabolism, growth, reproduction, and defense reactions. To this end information about the nutrient status has to flow from cell-to-cell, but also between distant organs via the long-distance transport tubes to trigger adaptive responses. This systemic signaling between roots and shoots is required to maintain mineral nutrient homeostasis in the different organs under varying environmental conditions. Recent results begin to shed light on the molecular components of the complex long-distance signaling pathways and it has been proposed that systemic signals can be transported through the xylem as well as via the phloem. Several molecules, including nutrients, hormones, sugars, and small RNAs have been suggested to be involved in systemic communication over long distance (Liu et al., 2009). Recent research has shown that in the case of mineral nutrients, the nutrients themselves, but also macromolecules like micro RNAs (miRNAs) can act as important information transmitters. The following review will summarize the current knowledge about phloem-mediated systemic signaling by miRNAs during ion nutrient allocation and adaptation to mineral nutrient deprivation, concentrating on the well-analyzed responses to a lack of potassium, sulfur, and copper.

## INTRODUCTION

Higher plants require at least 14 mineral elements that are vital for normal growth, development, and successful reproduction. Among these are macronutrients such as nitrogen, phosphorous, sulfur, magnesium, and calcium that are required in relatively large quantities (Maathuis, 2009; Ohkama-Ohtsu and Wasaki, 2010). In addition, small amounts of the microelements copper, chloride, boron, iron, manganese, molybdenum, nickel, and zinc are essential for all higher plants (Haensch and Mendel, 2009). Deprivation of even one of these macro- and microelements can cause growth retardation and other severe physiological disorders like chlorosis or necrosis, general symptoms that are characteristically associated with more than one mineral deficiency. Being sessile organisms, plants have to be capable of sensing and responding to variations in the availability of the different mineral nutrients in order to adapt to a wide range of environmental conditions. They evolved specialized organs that fulfil particular functions in nutrient and energy uptake, production, storage, and allocation. The root system, for example, is the major uptake site of minerals from soil, while the green shoot parts depend on mineral import and in return provide roots with organic nutrients assimilated during photosynthesis. The amount of mineral nutrients that can be absorbed by roots depends upon different factors like nutrient concentration, their adsorption to soil components, soil water content, and the size and uptake capacity of the root system. Once taken up into the plant body, the minerals are distributed between the different organs and allocated to growth, and storage, dependent on season, developmental stage, environmental conditions, and nutrient supply. The amount of nutrients that is consumed and can be stored differs significantly between plant organs, tissues, and even cell types (Conn and Gilliham, 2010). It has been proposed that the elemental distribution in plants is determined by the transport pathways as well as the storage properties of cells. The vacuoles that occupy most of the cell volume play a major role in the storage of mineral elements (Conn and Gilliham, 2010).

In natural environments, plants often have to face depletion or limited accessibility of one or more of the essential mineral elements, what can severely compromise their growth. The finite availability of inorganic nutrients is among the essential factors reducing crop productivity and yield in agriculture. On the other hand, elevated concentrations of certain minerals can be toxic to plant cells. Therefore, the amounts of different mineral nutrients have to be monitored in individual cells and organs and plants have developed a range of strategies to keep cytosolic ion concentrations within narrow ranges, in homeostasis, a state in which everything within the cell is in equilibrium and allows proper functioning. Homeostasis can, for example, be maintained and toxicity avoided by adjusting the compartmentation of minerals between cytosol and vacuoles.

To react to deprivation conditions, plants evolved a broad spectrum of diverse metabolic, physiological, and developmental adaptations. As an example, plants often allocate a greater proportion of their biomass to the root system when certain mineral nutrients are lacking.

A high number of components of the networks involved in mineral nutrient deficiency responses have meanwhile been identified, including transcription factors, riboregulators, and ubiquitin-related proteins (Rubio et al., 2009). Common and specific nutrient signaling pathways seem to exist and the responses can be highly complex (Schachtman and Shin, 2007). To integrate the reactions to nutrient deficiency at the whole-plant level, information about the local nutrient status of tissues, and organs needs to be communicated over short and long distances. When plants face nutrient deficiencies, the root system is normally the first organ to rapidly sense external deprivation. At such early stages, signals from roots can inform the shoot about upcoming deficits (Lough and Lucas, 2006; Liu et al., 2009). However, short periods of low nutrient supply will not immediately lead to deficiency within plant cells, as they can remobilize or regain nutrients from compartments like the vacuoles or mineral-containing molecules (Hammond et al., 2003). After longer starvation periods when shoots become deficient, signals from shoots can claim higher nutrient demands in order to increase uptake by the root system (Lough and Lucas, 2006; Liu et al., 2009).

## SYSTEMIC COMMUNICATION VIA THE PHLOEM

The exchange of water, nutrients, and small and large information molecules between the often distant organs occurs through the vasculature that pervades the whole-plant body and thus connects almost all plant parts. The vascular tissue consists of the tube systems of xylem and phloem that mediate long-distance transport of water, nutrients, metabolites, and small and large signaling molecules. Research during the past years has shown that the phloem is involved in long-distance signaling during many developmental processes and environmental responses. For example, the famous “florigen” (Chailakhyan, 1936), the almost universal factor(s) influencing flowering time over long-distance, is a classic case of phloem-mediated signaling from the leaves to the shoot apex (Zeevaart, 2008). Systemic acquired resistance (SAR), a broad-spectrum resistance protecting plants against repeated pathogen attacks, is another well-known example (Sticher et al., 1997). Also the systemic propagation of gene silencing seems to follow a similar phloem-mediated pathway (Palauqui et al., 1997; Voinnet and Baulcombe, 1997). Recent results moreover indicate that the phloem signaling network is also essential for communicating the nutrient status of different organs in order to coordinate supply with demand (Forde, 2002; Schachtman and Shin, 2007; Giehl et al., 2009; Chiou and Lin, 2011). Therefore, the phloem tube system is a key element for the coordination of a number of developmental and adaptation events that rely on an integrated response of the whole-plant on a systemic level. A variety of small molecules like nutrients themselves, phytohormones, and sugars, but recently also macromolecules could be related to systemic signaling processes during nutrient allocation (Liu et al., 2009).

## THE ROLE OF RNAs IN SYSTEMIC SIGNALING

The recent discovery that also macromolecules like proteins and RNAs can be transported through the phloem and may function as mobile signals (Lough and Lucas, 2006) significantly extends the potential complexity and possibilities of phloem-based information transfer. Indeed, endogenous RNA molecules were identified in phloem sap more than 40 years ago (Kollmann et al., 1970; Ziegler, 1975), but these “traces” of nucleic acids were initially considered to be a contamination from neighboring cells rather than an authentic constituent of the phloem transport fluid. This view was based on the assumption that RNA molecules have to act cell-autonomous and function solely at the site of their synthesis. However, recent results suggest that the phloem RNA population might have specific signaling or regulatory functions. Meanwhile thousands of phloem-specific transcripts (expressed sequence tags, ESTs; Omid et al., 2007) and non-coding RNAs of medium (Zhang et al., 2009) and small size (Yoo et al., 2004; Buhtz et al., 2008, 2010; Varkonyi-Gasic et al., 2010) could be identified in phloem samples from different plant species, including cucurbits, *Brassica napus*, and apple. Evidence accumulated over the past years is also supporting the notion that these RNA molecules are capable of long-distance movement and can probably act as regulatory molecules in a systemic signaling network operating at the whole-plant level (Lucas et al., 2001; Pant et al., 2008; Zhang et al., 2009; Buhtz et al., 2010 for a review see Lough and Lucas, 2006). Heterografting studies could, for instance, show that specific mRNA transcripts regulate developmental programs by moving long distance and inducing phenotypic alterations in their target tissues (Ruiz-Medrano et al., 1999; Kim et al., 2001; Haywood et al., 2005; Banerjee et al., 2006). The roles of small RNAs in the regulation of nutrient homeostasis will be discussed in the following paragraph.

## REGULATION OF NUTRIENT HOMEOSTASIS BY miRNA TRANSLOCATION

Especially regulatory micro RNAs (miRNAs) seem to play important roles during nutrient stress responses, as specific members of this class of small RNAs have been found to react to ion nutrient deficiencies. miRNAs are non-coding, endogenous small RNAs of 21–24 nt in length that contribute to the post-transcriptional regulation of gene expression by down-regulating the amounts of their complementary target transcripts. They are key regulators of plant development and are additionally involved in the orchestration of adaptive responses to various stress conditions (Bonnet et al., 2006; Mallory and Vaucheret, 2006; Kehr, 2012a,b; Sunkar et al., 2012; Baek et al., 2013). Among the well-analyzed miRNAs are miRNAs responsive to low phosphate (P), sulfate (S), or copper (Cu), respectively (Jones-Rhoades and Bartel, 2004; Sunkar and Zhu, 2004; Fujii et al., 2005; Bari et al., 2006; Chiou et al., 2006; Sunkar et al., 2006; Chiou, 2007; Yamasaki et al., 2007). The current knowledge of the roles of miRNAs in the systemic regulation of nutrient homeostasis will be discussed in the following paragraphs.

### PHOSPHATE NUTRITION

First indications that macromolecular signaling by miRNAs might be involved in the regulation of phosphate homeostasis came from the detailed characterization of the *pho2* mutant in *Arabidopsis thaliana* that over-accumulates Pi exclusively in shoots, caused by increased phosphate uptake and enhanced root-to-shoot translocation (Delhaize and Randall, 1995). Leaves even show signs of severe P toxicity when transpiration is high, characterized by chlorosis, and necrosis of mature leaf tips, suggesting that this mutation abolishes the regulation of phosphate homeostasis (Delhaize and Randall, 1995). The orthologous mutant in rice was named leaf tip necrosis1 (*ltn1*) due to its P toxicity phenotype (Hu et al., 2011). It has been found that the *pho2* mutant carries a mutation in the *UBC24* gene encoding an ubiquitin-conjugating E2 enzyme (Aung et al., 2006; Bari et al., 2006). It could as well be shown that *PHO2* is the target of miR399 (Aung et al., 2006; Bari et al., 2006). Overexpression of miR399 in transgenic plants phenocopied *pho2* mutants in that it also resulted in high shoot Pi levels in leaves and significantly reduced levels of the target transcript (Fujii et al., 2005; Bari et al., 2006; Hu et al., 2011). Meanwhile six members of the miR399 family have been described in *Arabidopsis* (ath-miR399a-f; miRBase19) that all seem to respond positively to phosphate deficiency, although not to the same extent (Bari et al., 2006). Eleven members of this miRNA family have been found in rice (miRBase19). In addition to the increase in shoot phosphate levels, heterologous overexpression of *Arabidopsis* miR399d in tomato led to an increased expression of P transporters and enhanced root proton exudation and thereby phosphate mobilization from soil (Gao et al., 2010). These observations imply that miR399 is a general regulator of phosphate allocation between roots, and shoots in different plant species, and can furthermore influence phosphate dissolution and uptake by the root system. Recently, miR399 was found to accumulate in phloem sap of different plant species under P deficiency, indicating a role in long-distance communication (Pant et al., 2008). This assumption was supported by reciprocal grafting experiments between miR399 overexpressing and wild-type plants grown under full nutrition, demonstrating that this miRNA can move from shoot to root, but not in the opposite direction. In addition, a reduction in the level of *PHO2* target mRNA in rootstocks could be observed, indicating that the translocated miR399 is functional (Lin et al., 2008; Pant et al., 2008). The same results were obtained when grafts between *Arabidopsis* wild-type plants and the miRNA processing mutant that carries a mutation in the RNA methylase HUA Enhancer 1 (*hen1-1*) were examined under phosphate deficient conditions (Buhtz et al., 2010). *hen1-1* mutants lack significant levels of mature miRNAs, caused by a mutation in the gene coding for the enzyme that methylates miRNA duplexes and thereby confers miRNA stability. It should be noted that the apparent unidirectional translocation of miR399 could be caused by the grafting system employing small seedlings that have very simple source–sink relations, with the root forming the only sink organ to which phloem assimilate transport will be directed (Buhtz et al., 2010).

A recent study identified an additional miRNA that increased upon phosphate limitation, named miR2111. This miRNA resembled the miR399 response in that it was almost undetectable under full nutrition, but became highly abundant during P limitation. Interestingly this miRNA, just like miR399, accumulated in phloem sap during phosphate limitation, indicating that it might as well be involved in long-distance communication (Pant et al., 2009). The confirmed target of miR2111 in *Arabidopsis* and soybean is a kelch repeat-containing F-box protein (Hsieh et al., 2009; Xu et al., 2013). F-box proteins are involved in the controlled degradation of cellular proteins *via* the ubiquitin pathway, like the target of miR399, *UBC24*. Therefore, an important role of posttranslational control of protein abundance under phosphate deprivation has been proposed (Hsieh et al., 2009).

### SULFUR DEPRIVATION

Another miRNA, miR395, was found to be involved in reacting to nutrient supply, in this case to insufficient sulfur: it strongly accumulates when sulfur levels decrease. Similar to miR399, also miR395 occurs in large families in most plant species (six members in *Arabidopsis*, 15 members in rice; miRBase19). miR395, was shown to target the two adenosine triphosphate ATP sulfurylases APS1 and APS4 and the low-affinity sulfate transporter SULTR2;1 (Jones-Rhoades and Bartel, 2004; Allen et al., 2005; Jones-Rhoades et al., 2006). APS are important for S assimilation, while SULTR2;1 is involved in S retrieval and translocation. It is striking that the expression pattern of miR395 resembles that of miR399, both being primarily expressed in vascular tissue (Aung et al., 2006). Like miR399 and miR2111, also miR395 has been found in phloem sap and is accumulating to high levels upon sulfur deficiency (Buhtz et al., 2008, 2010). Grafting experiments using the miRNA biosynthesis mutant *hen1-1* provided strong additional evidence that miR395 is indeed phloem-mobile and a negative effect of miR395 translocation on the expression of some of its target genes could be validated, suggesting that it can also act as a signaling molecule (Buhtz et al., 2010).

### COPPER DEFICIENCY

It was recently found that miR398 is involved in the post-transcriptional down-regulation of copper–zinc dismutase (*CSD1* and* CSD2*) expression during copper starvation, and this miRNA was shown to substantially increase during Cu deficiency (Yamasaki et al., 2007). miR398, and its complementarity to the three target mRNAs seems to be conserved in higher, but does not seem to exist in lower plants (Dugas and Bartel, 2008). miR398s, are occurring as comparably small families with only three members in *Arabidopsis* and two in rice (miRBase19). Expression of miR398s could be located to vascular tissues of leaves and roots in *Arabidopsis* plants (Sunkar et al., 2006; Dugas and Bartel, 2008). In addition to miR398 meanwhile more miRNAs, in particular miR397, miR408, and miR857 could be identified that were predicted to target different laccase and plantacyanin mRNAs, probably all reducing the amounts of these copper-containing proteins in favor of the essential photosynthesis protein plastocyanin (Fahlgren et al., 2007). The expression patterns of miR398b/c resemble that of miR395, and miR399. Also in accordance with miR395 and miR399, miR398 was detected in phloem samples and shown to strongly increase in this compartment in response to low Cu in *B. napus* plants (Buhtz et al., 2008). In addition to miR398 also miR397, and miR408 could be detected in phloem samples, and their levels were responsive to the amounts of available copper (Buhtz et al., 2010). This suggests that the regulation of copper homeostasis might have a systemic component, reflecting the proposed long-distance communication by miR395, and miR399 presumably involved in sulfur and phosphate allocation.

### NITRATE DEPRIVATION

In *Arabidopsis*, miRNAs are also involved in the regulation of nitrate transport: miR393 was induced by high nitrate and controls root architecture (Vidal et al., 2010), while miR169 is down-regulated under low nitrogen (Pant et al., 2009; Zhao et al., 2011). Both miRNAs were also found in phloem sap, where miR169 family members accumulated to higher levels than in leaves or roots (Buhtz et al., 2010). miR169, were shown to decrease in phloem sap under N limitation, what might indicate a role in shoot-to-root communication of the plant’s N status (Pant et al., 2009). However, whether any of the copper- or nitrate-responsive miRNAs, or any of the other phloem-localized RNAs (Buhtz et al., 2008, 2010; Pant et al., 2009; Varkonyi-Gasic et al., 2010), implicated with nutrient homeostasis are phloem-mobile in living plants has not been demonstrated yet and such a conclusion would require further experimentation.

## CONCLUDING REMARKS

**FIGURE 1:**
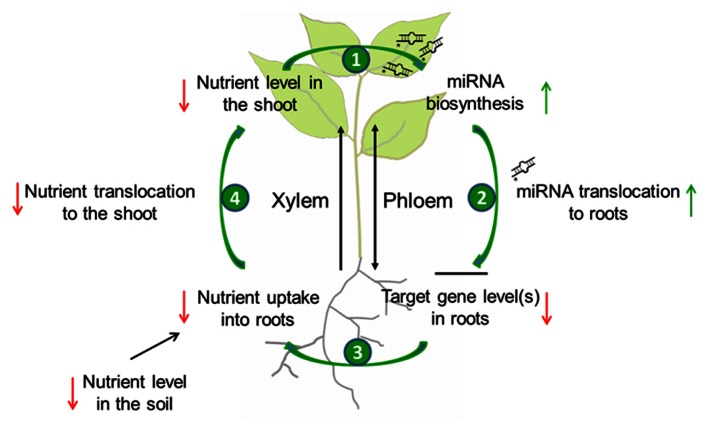
**Model of the possible role of miRNA translocation in the systemic regulation of nutrient homeostasis during limited supply**. When the level of a specific nutrient in the soil is low, nutrient uptake by roots as well as nutrient transport to the shoot through the xylem will decrease. A critically low level in the shoot will lead to the biosynthesis of nutrient-responsive miRNAs (1) that will be translocated to the root *via* the phloem (2). In the root, this miRNA will post-translationally reduce the amount of its target mRNA(s). As a consequence, nutrient uptake will be activated (3), and also nutrient allocation to the shoot will be elevated (4) what allows the plant to optimize shoot development under nutrient limitation.

The finding that specific miRNAs can transmit information regarding the nutrient status of plant organs over long distance was as such surprising. Indeed miRNAs were long believed to only act at the site of their transcription, in contrast to the mobile class of short interfering (si) RNAs that is supposed to be involved in the systemic spread of gene silencing (Alvarez et al., 2006; Valoczi et al., 2006; Dunoyer et al., 2007; Nogueira et al., 2009). Meanwhile grafting experiments provided convincing evidence that at least mature miR395, and miR399 are phloem-mobile, while other molecules (like miRNA precursors, hormones, or metabolites) could be excluded as systemic signals in these specific nutrient deficiency responses (Pant et al., 2008; Buhtz et al., 2010). However, a physiological necessity of miRNA translocation from shoot-to-root was questioned, because both mobile miRNAs can be synthesized in roots themselves (Aung et al., 2006; Kawashima et al., 2009) and an additional import therefore seems unnecessary. It has been proposed that the observed miRNA translocation might serve to rapidly communicate nutrient deprivation occurring in the shoot to the root, in order to trigger increased nutrient uptake by roots and the delivery to shoots. Such a mechanism would allow the plant to coordinate mineral homeostasis between organs, and to optimize shoot growth under nutrient limitation (Pant et al., 2008; Buhtz et al., 2010). A model of the role of the translocation of specific miRNAs in the systemic regulation of nutrient homeostasis is depicted in **Figure 1**.

Interestingly, many more miRNAs are found in phloem sap from different species, some accumulating in this compartment under nutrient stress similar to miR395 and miR399 (Buhtz et al., 2008, 2010; Pant et al., 2009; Varkonyi-Gasic et al., 2010). It is therefore likely that miRNA-mediated signaling *via* the phloem plays a more general role in nutrient deprivation responses. In addition to the nutrient dependent miRNAs, one other phloem-abundant miRNA, the development-related miR172, was meanwhile found to systemically influence tuber formation in grafting experiments when overexpressed, suggesting that it might as well constitute a phloem-mobile signal (Martin et al., 2009; Kasai et al., 2010). Given the numerous important regulatory functions of miRNAs, detailed transport studies will be invaluable to approve the *in vivo* mobility and the possible roles of additional miRNAs in systemic regulation.

## Conflict of Interest Statement

The author declares that the research was conducted in the absence of any commercial or financial relationships that could be construed as a potential conflict of interest.
